# Curvas del índice cintura-talla de adultos colombianos

**DOI:** 10.7705/biomedica.7647

**Published:** 2025-05-30

**Authors:** María Victoria Benjumea, Cristian Santa, Alejandro Estrada

**Affiliations:** 1 Escuela de Nutrición y Dietética, Universidad de Antioquia, Medellín, Colombia Universidad de Antioquia Escuela de Nutrición y Dietética Universidad de Antioquia Medellín Colombia; 2 Grupo de Investigación en Demografía y Salud, Universidad de Antioquia, Medellín, Colombia Universidad de Antioquia Grupo de Investigación en Demografía y Salud Universidad de Antioquia Medellín Colombia

**Keywords:** relación cintura-estatura, circunferencia de la cintura, antropometría, gráfico, adulto, Colombia, waist-height ratio, waist circumference, anthropometry, adult, charts, Colombia

## Abstract

**Introducción.:**

En Colombia, el sobrepeso y la obesidad abdominal se incrementan en forma progresiva en la población adulta, especialmente en las mujeres.

**Objetivo.:**

Elaborar curvas de los percentiles del índice cintura-talla de adultos colombianos por sexo y edad.

**Materiales y métodos.:**

Se realizó un análisis secundario de los datos de la Encuesta Nacional de la Situación Nutricional 2015, que contenía medidas de cintura, peso y talla en adultos entre los 20 y los 60 años. Se utilizaron modelos generalizados aditivos de localización, escala y forma, con transformación *box-cox power exponential* para construir las curvas de los percentiles. Se hizo una validación interna para garantizar que los modelos se ajustaran a los datos.

**Resultados.:**

Se estudiaron 23.759 adultos multiétnicos de Colombia, el 49,8 % fueron mujeres. Las curvas del índice cintura-talla de los hombres, se visualizaron con ligera curvatura, mientras que las de las mujeres aparecieron más planas. La mediana del índice se incrementó de forma continua en ambos sexos: hasta los 45 años en las mujeres (0,45 a 0,49) y hasta los 55 años en los hombres (0,44 a 0,49). En los hombres se mantuvo el valor de 0,50 después de los 55 años, pero en las mujeres no: permaneció en 0,50 hasta los 53 años y, de ahí en adelante, aumentó a 0,51.

**Conclusión.:**

Las curvas ajustadas con la distribución *box-cox power exponential* explicaron el comportamiento creciente del índice cintura-talla por edad y sexo y la capacidad predictiva del modelo. El incremento total de la mediana del índice por edad y sexo fue similar e incremental (mujeres: 0,45-0,51; hombres: 0,44-0,50).

## Abstract (en)

**Introduction.:**

Colombia presents a progressive increase in overweight and abdominal obesity in adults, with a higher impact on women.

**Objective.:**

To design percentile curves of the waist-height index of Colombian adults by sex and age.

**Materials and methods.:**

We did a secondary analysis of the data from the *Encuesta Nacional de la Situación Nutricional 2015*, which contained waist, weight, and height measurements of adults between 20 and 60 years of age. Generalized additive location, scale and shape additive models with box-cox power exponential transformation to construct the curves. An internal validation was performed to ensure the models fit the data.

**Results.:**

We studied 23,759 multiethnic adults from Colombia, 49.8% of whom were women. The waist-height index curves of men were visualized with slight curvature, while those of women appeared flatter. The median waist-height index increased continuously in both sexes: up to 45 years in women (0.45 to 0.49) and up to 55 years in men (0.44 to 0.49). In men, a value of 0.50 was maintained after 55 years, but not in women, since it remained at 0.50 until 53 years and thereafter increased to 0.51.

**Conclusion.:**

The curves fitted with the box-cox power exponential distribution explained the increasing behavior of the waist-height index by age and sex and the predictive capacity of the model. The total increase in the median of the waist-height index by age and sex was similar and incremental (women: 0.45-0.51; men: 0.44-0.50).

El número de personas obesas alrededor del mundo ya supera los mil millones. En el 2013, los estados miembros de la Asamblea Mundial de la Salud acordaron trabajar hacia un objetivo para el 2025, el de reducir en un 25,0 % la mortalidad por enfermedades no transmisibles y evitar que la prevalencia de obesidad o diabetes en adultos sobrepase los niveles del 2010. En el 2015, la Asamblea General de las Naciones Unidas adoptó la Agenda 2030 para el Desarrollo Sostenible con una serie de objetivos que incluyen: reducir en un tercio la mortalidad prematura por enfermedades no transmisibles y poner fin a “todas las formas de malnutrición” ^1^.

Colombia, como muchos de los países del planeta, presenta un incremento progresivo de las cifras de exceso de peso (sobrepeso y obesidad), al igual que de obesidad abdominal en la población adulta, con mayor impacto en las mujeres (exceso de peso en el 59,6 % de mujeres versus el 52,8 % de hombres; obesidad abdominal en el 59,6 % de mujeres versus el 39,3 % de hombres) ^2^. Esta información parte del análisis del índice de masa corporal (IMC), el cual , desafortunadamente, no aporta información sobre la composición corporal ni la ubicación de la grasa visceral del adulto. En otros estudios con población colombiana de diferentes etnias y condición económica, se han reportado datos absolutos de la circunferencia de la cintura, pero sin puntos de corte para tamizar el riesgo por edad y sexo ^2^^,^^3^.

Se ha publicado que la obesidad abdominal es un mejor indicador antropométrico del riesgo de enfermedad no transmisible que la obesidad general estimada mediante el IMC ^4^. Para determinar este riesgo, se calculan la circunferencia de la cintura y el índice cintura-talla ^5^^-^^7^.

En los últimos años, ha habido un aumento drástico en la prevalencia de la obesidad abdominal en Colombia ^2^^,^^8^, entre otros países de América ^9^. La obesidad representa un aumento de la grasa corporal y no solo del peso total. La grasa corporal estimada mediante absorciometría de rayos X de energía dual es el valor de referencia para evaluar la adiposidad; se correlaciona significativamente con el índice cintura-talla, pero requiere recursos costosos y no es factible en un entorno clínico y ambulatorio. Por tanto, es ideal contar con un marcador clínico válido y práctico de adiposidad, para establecer el riesgo de enfermedades no transmisibles en adultos ^4^. El IMC, la medida más utilizada y obtenida para diagnosticar obesidad, no proporciona información sobre la obesidad central o visceral, que es el principal factor determinante de las complicaciones cardiovasculares. De ahí, que algunos investigadores recomienden el uso conjunto del IMC y el índice cintura-talla ^10^ para predecir el riesgo cardiovascular.

La circunferencia de la cintura es un marcador de la obesidad central, pero depende de la edad, el sexo biológico y el origen étnico, lo que ha dificultado definir un único punto de corte de riesgo ^11^^,^^12^. El índice cintura-talla es un indicador que incorpora el tamaño corporal y la circunferencia de la cintura, tiene una buena correlación con la grasa visceral, predice las complicaciones de la obesidad ^13^^,^^14^ y se ha sugerido como un marcador de obesidad abdominal con un punto de corte uniforme según la edad y el sexo (hombres: 0,50, mujeres: 0,49) ^15^.

Varios investigadores han publicado sobre la capacidad predictiva del índice cintura-talla para enfermedades no transmisibles en adultos de distintas características y orígenes ^14^^,^^16^^-^^20^. Diversos autores han reportado que, al evaluar la obesidad en los adultos con el IMC, la circunferencia de la cintura y el índice cintura-talla, la prevalencia obtenida es mayor con el índice cintura-talla ^20^^,^^21^.

Ante lo planteado, se definió como objetivo de este estudio, construir curvas de percentiles del índice cintura-talla para adultos colombianos de ambos sexos, de todas las etnias y procedencias, que contribuyan a la detección oportuna de la obesidad abdominal para la prevención de enfermedades no transmisibles desde el nivel primario de atención en salud.

## Materiales y métodos

### Diseño del estudio y participantes

Se llevó a cabo un estudio transversal con los datos obtenidos en la Encuesta Nacional de la Situación Nutricional (ENSIN) del 2015 ^2^ de todos los adultos que contaban con las variables de interés para el estudio. Los detalles metodológicos de la ENSIN 2015 fueron publicados previamente ^22^. La ENSIN 2015 se diseñó para garantizar la representatividad de los residentes habituales del territorio nacional, diferenciando por cabecera municipal y áreas rurales. Se incluyeron seis regiones, 14 subregiones y 32 departamentos. La población incluida se seleccionó mediante un esquema de muestreo probabilístico, por conglomerados, estratificado y polietápico ^22^.

### Criterios de inclusión, exclusión y depuración de los datos

Los criterios de inclusión para el estudio fueron: adultos de ambos sexos entre los 20 y los 60 años con IMC normal (18,5-24,9 kg/m^2^), circunferencia de la cintura menor de 120 cm, talla mayor de 135 cm e índice cintura-talla menor de 0,7. Además, se agruparon los datos por edades simples y, en cada una, se calcularon los valores mínimos, los máximos y los percentiles correspondientes a los puntajes Z (-2 y +2) del índice de conicidad. Se evaluó el comportamiento creciente del índice de conicidad respecto a la edad y se estableció el dato atípico para la exclusión de datos como aquel valor por fuera del rango del puntaje Z (-2 y +2) en cada edad simple. La depuración y la limpieza de los datos atípicos redujeron el número de registros de la base de datos de 65.510 (todas las categorías de IMC) a 23.759 adultos con IMC normal. Los criterios de exclusión fueron embarazo o posparto inferior a tres meses en la fecha de la encuesta.

El estudio de origen de los datos ^22^ se llevó a cabo de acuerdo con las directrices establecidas en la Declaración de Helsinki y todos los participantes firmaron el consentimiento informado para aprobar su participación en la investigación. La base de datos estudiada no contaba con información que permitiera la identificación de los adultos.

### Fuente de los datos

La ENSIN 2015 obtuvo medidas antropométricas de todos los adultos del hogar, sin discapacidad física o mental, mediante encuestadoras capacitadas, entrenadas y supervisadas en la técnica antropométrica, quienes usaron instrumentos calibrados según las técnicas propuestas por Lohman *et al.*^23^ y por la *International Society for the Advancement of Kinanthropometry* (ISAK) para la circunferencia de la cintura ^24^. La talla se midió con el uso de un estadiómetro portátil con pieza móvil para cabeza (ShorrBoard^™^) con sensibilidad de un 1 mm. El peso corporal se obtuvo con una balanza Seca 874^™^ con capacidad de 200 kg y sensibilidad de más o menos 100 g para pesos de 0 a 50 kg y de más o menos 0,15 % para pesos superiores. La circunferencia de la cintura se midió en todos los adultos dos veces con una cinta antropométrica metálica (Rosscrafft^™^) con capacidad de 200 cm y sensibilidad de 1 mm. La medición se hizo paralela al piso con las personas en posición de pie, erguidas, sin zapatos, con las piernas separadas, los brazos doblados y abrazando el pecho, y la cabeza recta mirando al frente. La circunferencia de la cintura se midió mediante marcación en ambos lados de la persona a nivel de la línea media axilar, en el punto medio entre el reborde costal no flotante y el borde superior de la cresta ilíaca ^24^. El IMC se estimó como peso (kg) sobre talla al cuadrado (m^2^) y la normalidad para los adultos se definió con los puntos de corte establecidos por la Organización Mundial de la Salud (OMS): 18,5-24,9 kg/m^2 (^^3^.

### Análisis estadístico y modelo propuesto

El análisis descriptivo para cada una de las variables cuantitativas se agrupó por edad, sexo y etnia. Entre los análisis descriptivos, se tuvieron en cuenta promedios, medianas, desviaciones estándar, rangos intercuartílicos (IQR) y valor mínimo y máximo. Se evaluaron los comportamientos del índice cintura-talla según la edad mediante diagramas de distribución, tendencias y valores atípicos. Se elaboraron curvas suavizadas de tendencia central y de percentiles mediante métodos de regresión local. Además del comportamiento de la distribución, se evaluó el crecimiento o decrecimiento y la variabilidad de la circunferencia de la cintura por edad ^25^^,^^26^. Se hizo suavizamiento aditivo de regresión de cuantiles para controlar la variabilidad y observar la forma de la distribución de los datos de la circunferencia de la cintura ^27^.

Se partió de los modelos generalizados aditivos de localización, escala y forma (GAMLSS) ^27^^,^^28^ para analizar la relación del índice cintura-talla por sexo y edad en la población colombiana. Para detectar comportamientos no lineales, se aplicaron suavizadores penalizados ^29^ con términos aditivos como los polinomios fraccionales, de potencia ^29^ y las estrías cúbicas ^30^. Se usó el algoritmo de maximización no lineal vía máxima verosimilitud ^31^^,^^32^ para estimar los parámetros del modelo, los cuales se ajustaron contra una colección de distribuciones de probabilidad definida en los reales positivos para cuatro parámetros, tales como las familias *gamma* y *box-cox*^32^.

La base de datos de la ENSIN 2015 contenía información de la pertenencia étnica de los adultos y, por tanto, se realizaron análisis de armonización del ICT por edad, etnia y sexo, con la técnica de diferencias estandarizadas de sitio; se consideraron valores de diferencias estandarizadas de sitio dentro del rango de ±0,5 unidades de desviación estándar agrupadas, como tamaño mínimo del efecto para excluir los grupos poblacionales ^33^. La selección de los mejores modelos de las curvas tuvo en cuenta a aquellos con los menores valores, según los criterios generalizados (GAIC) y no generalizados (AIC) de Akaike ^33^^,^^34^ y *Global Deviance*^28^.

La normalidad de los residuales del modelo se evaluó con gráficas cuantil- cuantil (*QQ-plots*), prueba de Mardia ^35^ y pruebas estadísticas Q ^36^; el ajuste del modelo de crecimiento se evaluó mediante gráficos de gusano (*worm plots*) ^37^ y, para la especificación, se implementó la comparación de cuantiles ^29^ sobre las respuestas predichas con diferencias menores o iguales a 5,0 % respecto al percentil.

La función de enlace más significativa para los modelos del índice cintura-talla en la población estudiada fue la distribución *box-cox power exponential* de cuatro parámetros ^38^. La selección de los parámetros del modelo no lineal se obtuvo mediante el algoritmo cuasi-Newton con restricciones de límites ^39^.

La validación interna del modelo y su poder predictivo se llevaron a cabo mediante la comparación de cuantiles ^40^ teóricos sobre las respuestas con diferencias menores o iguales a 5,0 % respecto al valor del percentil obtenido. De esta manera, se establecieron las bandas de comportamiento suavizado para cada curva del índice cintura-talla por sexo y edad.

Todos los cálculos se hicieron con el *software* estadístico R^™^, versión 4.2.2. Los resultados se analizaron considerando un nivel de significancia del 5,0 % y la ecuación para estimar los percentiles del índice cintura-talla fue la siguiente:




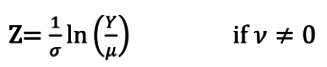




Donde:

Z = Z específica

σ = valor de la desviación estándar (parámetro sigma) del cuadro 2, para el sexo y la edad de la persona evaluada

ln = logaritmo neperiano

Y = valor del índice cintura/talla

μ = valor de la media (parámetro mu) del cuadro 2, para el sexo y la edad de la persona evaluada

v = valor de la asimetría (parámetro nu) del cuadro 2

**Cuadro 2:** Parámetros* y percentiles del índice cintura-talla de adultos colombianos por sexo y edad (n = 23.759)

Hombres																	
Edad	Parámetros				Percentil												
	Mu	Sigma	Nu	Tau	P _01_	P_2.3_	P_3_	P_10_	P_15.9_	P_25_	P_50_	P_75_	P_84.1_	P_90_	P_97_	P_97.7_	P_99.9_
20	0,44	0,07	-0,66	2,41	0,37	0,39	0,39	0,40	0,41	0,42	0,44	0,46	0,47	0,48	0,50	0,50	0,54
21	0,44	0,07	-0,41	2,48	0,37	0,39	0,39	0,41	0,41	0,42	0,44	0,46	0,47	0,48	0,50	0,50	0,54
22	0,44	0,07	-0,18	2,56	0,37	0,39	0,39	0,41	0,41	0,42	0,44	0,47	0,48	0,49	0,50	0,51	0,54
23	0,45	0,07	0,04	2,62	0,37	0,39	0,40	0,41	0,42	0,43	0,45	0,47	0,48	0,49	0,51	0,51	0,54
24	0,45	0,07	0,24	2,69	0,37	0,39	0,40	0,41	0,42	0,43	0,45	0,47	0,48	0,49	0,51	0,51	0,54
25	0,45	0,07	0,43	2,74	0,37	0,40	0,40	0,41	0,42	0,43	0,45	0,48	0,49	0,50	0,51	0,52	0,54
26	0,46	0,07	0,61	2,78	0,37	0,40	0,40	0,41	0,42	0,43	0,46	0,48	0,49	0,50	0,51	0,52	0,54
27	0,46	0,07	0,77	2,81	0,37	0,40	0,40	0,42	0,42	0,43	0,46	0,48	0,49	0,50	0,52	0,52	0,55
28	0,46	0,07	0,92	2,83	0,37	0,40	0,40	0,42	0,43	0,44	0,46	0,48	0,49	0,50	0,52	0,52	0,55
29	0,46	0,07	1,06	2,85	0,38	0,40	0,40	0,42	0,43	0,44	0,46	0,49	0,50	0,50	0,52	0,52	0,55
30	0,46	0,07	1,19	2,86	0,38	0,40	0,41	0,42	0,43	0,44	0,46	0,49	0,50	0,51	0,52	0,53	0,55
31	0,47	0,07	1,30	2,86	0,38	0,40	0,41	0,42	0,43	0,44	0,47	0,49	0,50	0,51	0,52	0,53	0,55
32	0,47	0,07	1,40	2,87	0,38	0,40	0,41	0,42	0,43	0,44	0,47	0,49	0,50	0,51	0,53	0,53	0,55
33	0,47	0,07	1,49	2,87	0,38	0,41	0,41	0,43	0,44	0,45	0,47	0,49	0,50	0,51	0,53	0,53	0,55
34	0,47	0,07	1,56	2,88	0,38	0,41	0,41	0,43	0,44	0,45	0,47	0,50	0,51	0,51	0,53	0,53	0,56
35	0,47	0,07	1,62	2,88	0,38	0,41	0,41	0,43	0,44	0,45	0,47	0,50	0,51	0,52	0,53	0,53	0,56
36	0,48	0,07	1,67	2,89	0,38	0,41	0,41	0,43	0,44	0,45	0,48	0,50	0,51	0,52	0,53	0,54	0,56
37	0,48	0,07	1,72	2,89	0,38	0,41	0,41	0,43	0,44	0,45	0,48	0,50	0,51	0,52	0,53	0,54	0,56
38	0,48	0,07	1,75	2,90	0,38	0,41	0,41	0,43	0,44	0,45	0,48	0,50	0,51	0,52	0,54	0,54	0,56
39	0,48	0,07	1,78	2,90	0,38	0,41	0,42	0,43	0,44	0,45	0,48	0,50	0,51	0,52	0,54	0,54	0,56
40	0,48	0,07	1,80	2,91	0,38	0,41	0,42	0,43	0,44	0,46	0,48	0,51	0,52	0,52	0,54	0,54	0,56
41	0,48	0,07	1,82	2,92	0,38	0,41	0,42	0,44	0,44	0,46	0,48	0,51	0,52	0,52	0,54	0,54	0,57
42	0,48	0,07	1,84	2,92	0,38	0,41	0,42	0,44	0,45	0,46	0,48	0,51	0,52	0,53	0,54	0,54	0,57
43	0,48	0,07	1,86	2,92	0,38	0,42	0,42	0,44	0,45	0,46	0,48	0,51	0,52	0,53	0,54	0,55	0,57
44	0,49	0,07	1,87	2,92	0,39	0,42	0,42	0,44	0,45	0,46	0,49	0,51	0,52	0,53	0,54	0,55	0,57
45	0,49	0,07	1,88	2,92	0,39	0,42	0,42	0,44	0,45	0,46	0,49	0,51	0,52	0,53	0,55	0,55	0,57
46	0,49	0,07	1,89	2,91	0,39	0,42	0,42	0,44	0,45	0,46	0,49	0,51	0,52	0,53	0,55	0,55	0,57
47	0,49	0,07	1,90	2,90	0,39	0,42	0,42	0,44	0,45	0,46	0,49	0,51	0,52	0,53	0,55	0,55	0,57
48	0,49	0,07	1,90	2,88	0,39	0,42	0,42	0,44	0,45	0,46	0,49	0,51	0,53	0,53	0,55	0,55	0,58
49	0,49	0,07	1,91	2,87	0,39	0,42	0,42	0,44	0,45	0,46	0,49	0,52	0,53	0,53	0,55	0,55	0,58
50	0,49	0,07	1,91	2,85	0,39	0,42	0,42	0,44	0,45	0,46	0,49	0,52	0,53	0,54	0,55	0,56	0,58
51	0,49	0,07	1,91	2,83	0,39	0,42	0,42	0,44	0,45	0,47	0,49	0,52	0,53	0,54	0,55	0,56	0,58
52	0,49	0,07	1,91	2,81	0,39	0,42	0,42	0,44	0,45	0,47	0,49	0,52	0,53	0,54	0,55	0,56	0,58
53	0,49	0,07	1,91	2,79	0,39	0,42	0,42	0,44	0,45	0,47	0,49	0,52	0,53	0,54	0,56	0,56	0,58
54	0,49	0,07	1,91	2,77	0,39	0,42	0,42	0,44	0,46	0,47	0,49	0,52	0,53	0,54	0,56	0,56	0,59
55	0,50	0,07	1,90	2,75	0,39	0,42	0,42	0,45	0,46	0,47	0,50	0,52	0,53	0,54	0,56	0,56	0,59
56	0,50	0,07	1,90	2,72	0,39	0,42	0,43	0,45	0,46	0,47	0,50	0,52	0,53	0,54	0,56	0,56	0,59
57	0,50	0,07	1,90	2,70	0,39	0,42	0,43	0,45	0,46	0,47	0,50	0,52	0,54	0,54	0,56	0,56	0,59
58	0,50	0,07	1,90	2,68	0,39	0,42	0,43	0,45	0,46	0,47	0,50	0,52	0,54	0,55	0,56	0,57	0,59
59	0,50	0,07	1,89	2,66	0,38	0,42	0,43	0,45	0,46	0,47	0,50	0,53	0,54	0,55	0,56	0,57	0,59
60	0,50	0,07	1,89	2,64	0,38	0,42	0,43	0,45	0,46	0,47	0,50	0,53	0,54	0,55	0,56	0,57	0,60
**Mujeres**																	
**Edad**	**Parámetros**				**Percentil**												
	**Mu**	**Sigma**	**Nu**	**Tau**	**P**_**01**_	**P**_**2.3**_	**P**_**3**_	**P**_**10**_	**P**_**15.9**_	**P**_**25**_	**P**_**50**_	**P**_**75**_	**P**_**84.1**_	**P**_**90**_	**P**_**97**_	**P**_**97.7**_	**P**_**99.9**_
20	0,45	0,08	0,08	2,66	0,37	0,39	0,40	0,41	0,42	0,43	0,45	0,48	20	0,45	0,08	0,08	2,66
21	0,46	0,07	0,10	2,64	0,37	0,40	0,40	0,41	0,42	0,43	0,46	0,48	21	0,46	0,07	0,10	2,64
22	0,46	0,07	0,13	2,61	0,37	0,40	0,40	0,42	0,42	0,43	0,46	0,48	0,50	22	0,46	0,07	0,13
23	0,46	0,07	0,15	2,59	0,37	0,40	0,40	0,42	0,43	0,44	0,46	0,49	0,50	23	0,46	0,07	0,15
24	0,46	0,07	0,18	2,57	0,38	0,40	0,40	0,42	0,43	0,44	0,46	0,49	0,50	24	0,46	0,07	0,18
25	0,46	0,07	0,20	2,55	0,38	0,40	0,41	0,42	0,43	0,44	0,46	0,49	0,50	25	0,46	0,07	0,20
26	0,47	0,07	0,22	2,53	0,38	0,40	0,41	0,42	0,43	0,44	0,47	0,49	0,50	26	0,47	0,07	0,22
27	0,47	0,07	0,24	2,51	0,38	0,41	0,41	0,43	0,43	0,44	0,47	0,49	0,51	27	0,47	0,07	0,24
28	0,47	0,07	0,27	2,49	0,38	0,41	0,41	0,43	0,44	0,45	0,47	0,50	0,51	28	0,47	0,07	0,27
29	0,47	0,07	0,29	2,47	0,38	0,41	0,41	0,43	0,44	0,45	0,47	0,50	0,51	29	0,47	0,07	0,29
30	0,47	0,07	0,31	2,46	0,38	0,41	0,41	0,43	0,44	0,45	0,47	0,50	0,51	30	0,47	0,07	0,31
31	0,48	0,07	0,33	2,44	0,38	0,41	0,42	0,43	0,44	0,45	0,48	0,50	0,51	31	0,48	0,07	0,33
32	0,48	0,07	0,36	2,43	0,39	0,41	0,42	0,43	0,44	0,45	0,48	0,50	0,51	32	0,48	0,07	0,36
33	0,48	0,07	0,38	2,42	0,39	0,41	0,42	0,44	0,44	0,45	0,48	0,50	0,52	33	0,48	0,07	0,38
34	0,48	0,07	0,40	2,41	0,39	0,42	0,42	0,44	0,45	0,46	0,48	0,51	0,52	34	0,48	0,07	0,40
35	0,48	0,07	0,42	2,40	0,39	0,42	0,42	0,44	0,45	0,46	0,48	0,51	0,52	35	0,48	0,07	0,42
36	0,48	0,07	0,44	2,40	0,39	0,42	0,42	0,44	0,45	0,46	0,48	0,51	0,52	36	0,48	0,07	0,44
37	0,48	0,07	0,47	2,40	0,39	0,42	0,42	0,44	0,45	0,46	0,48	0,51	0,52	37	0,48	0,07	0,47
38	0,49	0,07	0,49	2,40	0,39	0,42	0,42	0,44	0,45	0,46	0,49	0,51	0,52	38	0,49	0,07	0,49
39	0,49	0,07	0,51	2,40	0,39	0,42	0,43	0,44	0,45	0,46	0,49	0,51	0,52	39	0,49	0,07	0,51
40	0,49	0,07	0,53	2,40	0,39	0,42	0,43	0,44	0,45	0,46	0,49	0,51	40	0,49	0,07	0,53	2,40
41	0,49	0,07	0,55	2,41	0,39	0,42	0,43	0,45	0,45	0,47	0,49	0,52	41	0,49	0,07	0,55	2,41
42	0,49	0,07	0,57	2,41	0,40	0,43	0,43	0,45	0,46	0,47	0,49	0,52	42	0,49	0,07	0,57	2,41
43	0,49	0,07	0,59	2,42	0,40	0,43	0,43	0,45	0,46	0,47	0,49	0,52	43	0,49	0,07	0,59	2,42
44	0,49	0,07	0,61	2,43	0,40	0,43	0,43	0,45	0,46	0,47	0,49	0,52	44	0,49	0,07	0,61	2,43
45	0,50	0,07	0,64	2,44	0,40	0,43	0,43	0,45	0,46	0,47	0,50	0,52	45	0,50	0,07	0,64	2,44
46	0,50	0,07	0,66	2,45	0,40	0,43	0,43	0,45	0,46	0,47	0,50	0,52	46	0,50	0,07	0,66	2,45
47	0,50	0,07	0,68	2,47	0,40	0,43	0,43	0,45	0,46	0,47	0,50	0,52	47	0,50	0,07	0,68	2,47
48	0,50	0,07	0,70	2,48	0,40	0,43	0,43	0,45	0,46	0,47	0,50	0,52	48	0,50	0,07	0,70	2,48
49	0,50	0,07	0,72	2,50	0,40	0,43	0,43	0,45	0,46	0,47	0,50	0,53	49	0,50	0,07	0,72	2,50
50	0,50	0,07	0,74	2,51	0,40	0,43	0,43	0,45	0,46	0,47	0,50	0,53	50	0,50	0,07	0,74	2,51
51	0,50	0,07	0,76	2,53	0,40	0,43	0,44	0,45	0,46	0,48	0,50	0,53	51	0,50	0,07	0,76	2,53
52	0,50	0,07	0,78	2,55	0,40	0,43	0,44	0,45	0,46	0,48	0,50	0,53	52	0,50	0,07	0,78	2,55
53	0,50	0,07	0,80	2,56	0,40	0,43	0,44	0,46	0,47	0,48	0,50	0,53	53	0,50	0,07	0,80	2,56
54	0,51	0,08	0,82	2,58	0,40	0,43	0,44	0,46	0,47	0,48	0,51	0,53	54	0,51	0,08	0,82	2,58
55	0,51	0,08	0,84	2,59	0,40	0,43	0,44	0,46	0,47	0,48	0,51	0,53	55	0,51	0,08	0,84	2,59
56	0,51	0,08	0,86	2,61	0,40	0,43	0,44	0,46	0,47	0,48	0,51	0,54	56	0,51	0,08	0,86	2,61
57	0,51	0,08	0,88	2,63	0,40	0,43	0,44	0,46	0,47	0,48	0,51	0,54	57	0,51	0,08	0,88	2,63
58	0,51	0,08	0,90	2,64	0,40	0,44	0,44	0,46	0,47	0,48	0,51	0,54	58	0,51	0,08	0,90	2,64
59	0,51	0,08	0,92	2,66	0,40	0,44	0,44	0,46	0,47	0,48	0,51	0,54	59	0,51	0,08	0,92	2,66
60	0,51	0,08	0,94	2,68	0,40	0,44	0,44	0,46	0,47	0,48	0,51	0,54	60	0,51	0,08	0,94	2,68

* Modelo GAMLSS (modelo aditivo generalizado para ubicación, escala y forma) con transformación *box-cox power exponential*

Mu: media; Sigma: desviación estándar; Nu: asimetría; Tau: curtosis

## Resultados

Se estudiaron 23.759 adultos entre los 20 y los 60 años de edad, de todas las etnias y lugares de residencia en Colombia; el 49,8 % (n = 11.828) fueron mujeres. La mayor proporción de adultos correspondió a aquellos sin ninguna pertenencia étnica (83,1 %; n = 19.750), seguida de las etnias «negros, afrodescendientes, raizales y palenqueros» (8,8 %; n = 2.080) e indígenas (8,1 %; n = 1.929). La descripción de la población según las variables estudiadas se presenta en el cuadro 1. Entre los hombres, la mediana de la talla fue mayor en los negros, afrodescendientes, raizales y palenqueros y aquellos sin pertenencia étnica, la mediana de la cintura fue más alta en los adultos sin pertenencia étnica y los indígenas, y el índice cintura-talla fue ligeramente superior en los indígenas. Entre las mujeres, las medianas de cintura y talla fueron ligeramente superiores en las afrodescendientes, raizales y palenqueras y la mediana del índice cintura-talla fue más alta en las indígenas (cuadro 1).

**Cuadro 1:** Descripción de la población estudiada según las variables de interés (N = 23.759)

Etnia	Variable	Hombres (n = 11.931)								
		n	Mu	Sigma	IQR	Mín.	P25	P50	P75	Máx.
Indígena	Cintura	1002	77,55	7,88	5,68	63,70	73,50	**77,50**	81,38	95,00
Indígena	Edad	1002	35,29	21,25	11,78	20,00	24,58	**32,17**	45,83	59,92
Indígena	ICT	1002	0,48	0,05	0,03	0,39	0,45	**0,48**	0,50	0,58
Indígena	IMC	1002	22,64	2,37	1,59	18,56	21,58	**22,84**	23,94	25,00
Indígena	Peso	1002	59,53	8,50	6,11	37,90	55,20	**59,30**	63,70	81,40
Indígena	Talla	1002	162,07	8,50	6,44	138,40	157,70	**161,90**	166,20	184,20
NARP	Cintura	1138	77,76	8,80	6,09	63,10	73,20	**77,10**	82,00	97,20
NARP	Edad	1138	35,78	19,42	11,58	20,00	25,83	**33,25**	45,25	59,92
NARP	ICT	1138	0,46	0,05	0,04	0,36	0,43	**0,45**	0,48	0,57
NARP	IMC	1138	22,37	2,56	1,66	18,51	21,19	**22,48**	23,75	25,00
NARP	Peso	1138	65,13	10,00	7,11	43,00	60,00	**64,95**	70,00	90,00
NARP	Talla	1138	170,51	9,20	7,09	145,10	165,80	**170,50**	175,00	194,60
Ninguno	Cintura	9791	78,99	8,90	6,10	62,00	74,50	**78,90**	83,40	98,10
Ninguno	Edad	9791	35,60	20,58	11,78	20,00	25,00	**32,92**	45,58	59,92
Ninguno	ICT	9791	0,47	0,05	0,04	0,36	0,44	**0,47**	0,50	0,60
Ninguno	IMC	9791	22,43	2,71	1,68	18,50	21,14	**22,64**	23,85	25,00
Ninguno	Peso	9791	63,47	9,40	6,76	42,20	58,70	**63,40**	68,10	89,20
Ninguno	Talla	9791	168,12	8,90	6,73	142,20	163,60	**168,20**	172,50	199,50
**Etnia**	**Variable**	**Mujeres (n=11.828)**								
		**n**	**Mu**	**Sigma**	**IQR**	**Mín.**	**P25**	**P50**	**P75**	**Máx.**
Indígena	Cintura	927	74,58	7,60	5,67	58,20	70,60	**74,20**	78,20	91,00
Indígena	Edad	927	34,80	19,04	11,57	20,00	24,75	**32,00**	43,79	59,92
Indígena	ICT	927	0,49	0,06	0,04	0,38	0,47	**0,49**	0,52	0,63
Indígena	IMC	927	22,54	2,66	1,69	18,52	21,33	**22,78**	23,99	25,00
Indígena	Peso	927	51,46	7,95	5,80	35,80	47,25	**51,40**	55,20	74,50
Indígena	Talla	927	150,97	8,10	6,24	135,40	146,60	**150,90**	154,70	172,80
NARP	Cintura	942	74,90	8,00	5,68	60,80	70,80	**75,00**	78,80	91,00
NARP	Edad	942	34,73	17,23	10,89	20,00	25,44	**32,50**	42,67	59,83
NARP	ICT	942	0,47	0,05	0,04	0,38	0,45	**0,47**	0,50	0,59
NARP	IMC	942	22,52	2,64	1,68	18,53	21,31	**22,78**	23,95	24,99
NARP	Peso	942	57,01	8,20	6,09	38,20	52,80	**57,05**	61,00	77,50
NARP	Talla	942	159,00	8,50	6,24	139,50	154,80	**158,85**	163,30	178,70
Ninguno	Cintura	9959	75,00	8,00	5,76	58,50	71,00	**74,80**	79,00	94,50
Ninguno	Edad	9959	35,21	18,58	11,24	20,00	25,42	**32,83**	44,00	59,92
Ninguno	ICT	9959	0,48	0,05	0,04	0,37	0,45	**0,48**	0,51	0,62
Ninguno	IMC	9959	22,51	2,66	1,69	18,50	21,27	**22,76**	23,94	25,00
Ninguno	Peso	9959	55,05	7,95	5,87	36,50	51,00	**55,00**	58,95	77,80
Ninguno	Talla	9959	156,30	8,30	6,28	135,50	152,00	**156,20**	160,30	186,80

Ninguno: no se identifica como perteneciente a ninguna etnia; NARP: negro, afrodescendiente, raizal o palenquero; ICT: índice de cintura-talla; IMC: índice de masa corporal; Mu: media; Sigma: desviación estándar; IQR: rango intercuartílico; Mín.: mínimo; Máx.: máximo

**Figura 1:**
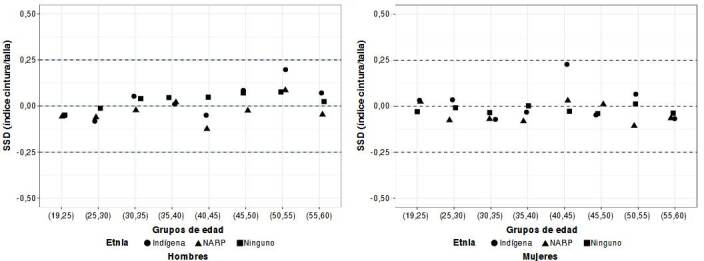
Diferencias estandarizadas por sitio del índice cintura-talla de los adultos evaluados, según etnia, edad y sexo. Izquierda: hombres, derecha: mujeres.

Después de evaluar el comportamiento del índice cintura-talla por etnia, sexo y edad, no se encontraron diferencias significativas por sitio en los adultos por etnia, por lo que no se consideró esta variable al diseñar cada modelo (figura 1).

En lo correspondiente a la mediana del índice cintura-talla, el incremento por sexo fue similar; en las mujeres entre los 20 y los 60 años fue de 0,45 a 0,51 y, en los hombres de las mismas edades, fue de 0,44 a 0,50 (cuadro 2).

**Figura 2:**
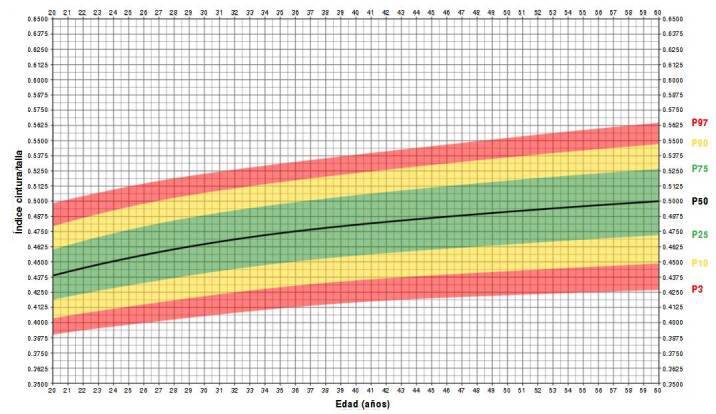
Curvas de percentiles del índice cintura-talla de hombres adultos colombianos por edad (n = 11.931)

**Figura 3:**
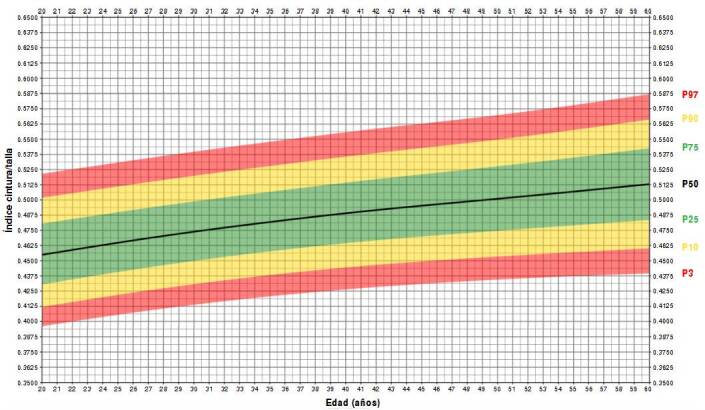
Curvas de percentiles del índice cintura-talla de mujeres adultas colombianas por edad (n = 11.828)

Las curvas del índice cintura-talla para los adultos colombianos mostraron comportamientos diferentes por edad y sexo (figuras 2 y 3, y cuadro 2). Las curvas de percentiles de los hombres muestran una ligera curvatura (figura 2), mientras que, las de las mujeres se ven más planas (figura 3). El valor del incremento percentil del índice cintura-talla fue ligeramente superior en los distintos canales de la curva de las mujeres (figura 3). La mediana del índice cintura-talla aumentó de forma continua hasta los 45 años en las mujeres (0,45 a 0,49) y, en los hombres, hasta los 55 años (0,44 a 0,49) (figuras 2 y 3 y cuadro 2). En los hombres, se mantuvo el mismo valor de 0,50 después de los 55 años (figura 2), pero en las mujeres fue diferente, ya que se mantuvo en 0,50 hasta los 53 años y, de ahí en adelante, aumentó a 0,51 (figura 3).

En cuanto al percentil 75, el incremento del índice cintura-talla en las mujeres fue de 0,48 a 0,49 hasta los 28 años, pero en los hombres fue de 0,46 a 0,49 hasta los 33 años. De ahí en adelante, en ambos sexos, fue de 0,50 o más. Solo en el percentil 90 de la curva de las mujeres, se encontró un índice cintura-talla de 0,50 desde los 20 años que aumentó hasta 0,57 a los 60 años (figura 3, cuadro 2). En los hombres, esta situación se observó a partir de los 25 años, con un aumento del índice cintura-talla hasta 0,55 a los 60 años (figura 2, cuadro 2).

Para facilitar el uso y la aplicación de la ecuación obtenida para estimar los percentiles del índice cintura-talla, se diseñó el siguiente ejemplo:

En una mujer con IMC normal, se calculó el índice cintura-talla a los 50 años y su valor fue de 0,485. Se revisó el cuadro del índice cintura-talla de mujeres (cuadro 2) y se ubicó la fila de la edad de 50 años. Los parámetros obtenidos fueron los siguientes: μ = 0,5, σ = 0,07, v = 0,74 y t = 2,51. Dado que v ≠ 0, entonces el puntaje z correspondió a:

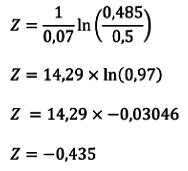




El puntaje z de -0,435 resultó en una probabilidad del 33,18 %, lo que indicó que el índice de cintura-talla estuvo en el canal comprendido entre los percentiles P_25_ y P_50_.

Finalmente, en la validación interna del modelo, se compararon los percentiles teóricos de la curva del índice cintura-talla por edad en hombres y mujeres. Esta comparación permitió evaluar la proporción de datos utilizados para el ajuste del modelo por debajo de cada percentil teórico. Para los dos modelos estimados de curvas, se contó con un buen ajuste, así que, las diferencias en cada percentil no superaron el 1,0 % de la información. Por tanto, las curvas del índice cintura-talla para la edad, ajustadas con la distribución *box-cox power exponential*, permitieron explicar el comportamiento creciente del índice cintura-talla y probar la capacidad predictiva del modelo (cuadro 3).

**Cuadro 3:** Validación interna del modelo por sexo

**Modelo *box-cox power exponential***					
Hombres			Mujeres		
Percentil	Predicho %	Diferencia %	Percentil	Predicho %	Diferencia %
3	3,21	0,21	3	3,21	0,21
10	10,01	0,01	10	10,09	0,09
25	24,79	0,21	25	24,72	0,28
50	50,13	0,13	50	50,00	0,00
75	75,18	0,18	75	75,51	0,51
90	89,94	0,06	90	89,63	0,37
97	96,93	0,07	97	96,88	0,12

## Discusión

La identificación de predictores de riesgo de enfermedades no transmisibles desde el nivel primario de atención es útil y necesaria para los profesionales de la salud, en especial en Colombia, donde la obesidad abdominal en adultos va en aumento, existe una gran diversidad étnica y las mediciones antropométricas requeridas se toman de forma rutinaria en la consulta médica y nutricional. Por lo tanto, las curvas de percentiles del índice cintura-talla por edad, sexo y etnia para Colombia -propuestas en este estudio- son una contribución novedosa e importante para la salud nutricional de la población adulta.

Disfunciones metabólicas como la obesidad, la resistencia a la insulina, el síndrome metabólico y la tolerancia a la glucosa están fuertemente relacionadas entre sí y el IMC no es el indicador antropométrico adecuado para detectarlas ^41^^-^^43^. La presencia de cualquiera de esas alteraciones metabólicas se traduce en un gran riesgo de padecer enfermedades cardiovasculares y diabetes ^14^^,^^44^. Las mediciones antropométricas, como algunas circunferencias corporales, los pliegues de grasa y el índice cintura-talla se han utilizado ampliamente en el estudio de distintas enfermedades no transmisibles. El índice cintura-talla tiene una ventaja sobre el IMC y es que proporciona información sobre la distribución central de la grasa corporal, particularmente, de la grasa abdominal o visceral ^20^^,^^45^. La distribución central de la grasa se asocia con riesgos cardio-metabólicos más significativos que la grasa corporal total como lo demuestran varias publicaciones ^4^^,^^16^.

La evaluación de la obesidad abdominal con el índice cintura-talla en el nivel primario de atención en salud es una práctica útil para identificar personas con IMC normal, pero con características metabólicas que sugieren obesidad ^5^^,^^45^. De esta manera, el contar con curvas del índice cintura-talla por edad y sexo permitirá hacerlo de forma rutinaria en la consulta de salud y nutrición.

Entre la evidencia publicada sobre el tema, se encuentra el estudio de Ramírez-Vélez *et al.*^46^, quienes analizaron los datos de la ENSIN 2010 ^8^. En este reporte se elaboraron curvas ROC para evaluar el punto de corte óptimo del índice cintura-talla para adultos, según las definiciones de la OMS ^47^. Similar a los resultados de este estudio, las medianas del índice cintura-talla aumentaron con la edad en ambos sexos. Sin embargo, llama la atención que, tanto la mediana por edad del índice cintura-talla de las mujeres a partir de los 25 años, como la de los hombres a partir de los 30 años, superan el valor de 0,50. Esto indicaría que la mayor parte de la población colombiana está en riesgo de padecer enfermedades no transmisibles ^46^^-^^48^.

Al revisar con detalle las dos curvas del índice cintura-talla publicadas por Ramírez-Vélez *et al.,* estas no concuerdan con los datos anteriores, pues la mediana del índice cintura-talla en mujeres (0,50) se observa después de los 55 años y, en los hombres, después de los 45 años ^46^. Además, en el artículo mencionado, el índice cintura-talla igual o superior a 0,50 se presenta en el percentil 25 a partir de los 40 años en los hombres y a partir de los 35 años en las mujeres.

La razón principal para la discordancia entre los datos de Ramírez-Vélez *et al.* y los resultados de este estudio, es que, para el diseño de las curvas de percentiles aquí propuestas, se trabajó solo con adultos con IMC normal para promover el tamizaje precoz de la obesidad abdominal en la consulta.

Según la propuesta de Ramírez-Vélez *et al.*^46^, que establece puntos de corte por sexo para estimar sobrepeso (mujeres: 0,536, hombres: 0,521) y obesidad (mujeres: 0,587, hombres: 0,579), las curvas del índice cintura-talla del presente estudio indican riesgo de sobrepeso para mujeres y hombres del percentil 75 (P_75_), a partir de los 49 años, y riesgo de obesidad para las mujeres del percentil 97 (P_97_) a partir de los 54 años y para los hombres del percentil 97,7 (P_97,7_) a partir de los 58 años.

Como ya se describió, en Colombia, el exceso de peso y la obesidad abdominal son cada vez más frecuentes en los adultos, razón por la cual los datos obtenidos en la ENSIN 2010 ^8^ y la de 2015 ^2^^,^^8^ muestran prevalencias diferentes y crecientes. El análisis realizado al estudio de Ramírez-Vélez *et al.*^46^ y la transición nutricional en el país motivaron la elaboración de las curvas del índice cintura-talla del presente trabajo a partir de mediciones más recientes de adultos con IMC normal. Estas curvas serán validadas en una segunda fase con marcadores clínicos de adultos para definir si el punto de corte internacionalmente propuesto para el índice cintura-talla (0,50 para ambos sexos) se ajusta o no a la población multiétnica colombiana ^3^^,^^16^^,^^17^^,^^44^^,^^49^^-^^51^.

Otros autores que han diseñado curvas del índice cintura-talla son López- Legarrea *et al.*, a partir de datos de población chilena adulta ^49^. En este trabajo, se describió que todos los grupos de edad presentaban valores de índice cintura-talla superiores a 0,5, lo que indica, según los autores, la existencia de riesgo metabólico en la mayoría de la población. El valor medio del índice cintura-talla fue significativamente mayor en las mujeres (p < 0,01) que en los hombres (p < 0,001), con excepción del grupo de edad más joven, en el que no hubo diferencias estadísticamente significativas ^49^.

Respecto al índice de cintura-talla en población con sobrepeso, se obtuvo que, en los hombres, el punto de corte fue de 0,53 con sensibilidad del 84,6 % y especificidad del 81,7 % y, en las mujeres, fue de 0,55 con sensibilidad del 82,8 % y especificidad del 87,1 %. En la población con obesidad, el punto de corte en los hombres fue de 0,59 con sensibilidad del 86,6 % y especificidad del 85,0 %; en las mujeres, se obtuvo el mismo punto de corte (0,59) pero con sensibilidad del 88,2 % y especificidad del 80,9 %.

Al relacionar los puntos de corte de sobrepeso (mujeres: 0,55, hombres: 0,53) y obesidad (mujeres: 0,59, hombres: 0,59) del estudio chileno ^49^ con los datos de la población colombiana aquí evaluada, se encontró que las mujeres con sobrepeso serían aquellas del percentil 84,1 (P_84,1_) a partir de los 54 años y los hombres con sobrepeso serían los del percentil 75 (P_75_) a partir de los 59 años; las mujeres con obesidad serían las del percentil 97 (P_97_) a partir de los 59 años y los hombres con obesidad serían los del percentil 99,9 (P_99,9_) a partir de los 54 años. La aplicación de estos puntos de corte - definidos por López-Legarrea *et al.*- llevarían a diagnósticos e intervenciones tardíos en nuestra población.

A pesar de no encontrar a la fecha otros estudios que hayan publicado curvas del índice cintura-talla para adultos, los resultados de este trabajo se interpretaron con puntos de corte de riesgo de enfermedades no transmisibles identificados en otros estudios. El primero es el reportado por la Encuesta Nacional de Salud y Nutrición (ENSANUT) de México en el 2016 ^52^, en el que se encontraron diferentes puntos de corte del índice cintura-talla para discriminar entre grupos con diferentes factores de riesgo metabólico (mujeres: 0,535 y hombres: 0,555). En la población mexicana, se encontró que el índice cintura-talla discrimina mejor la circunferencia de la cintura elevada y los triglicéridos altos en las mujeres, mientras que, en el grupo de los hombres, discrimina mejor la elevación de la glucosa en ayunas, la de la presión arterial y la de la circunferencia de la cintura. Al ubicar esos puntos de corte en las curvas diseñadas con datos de adultos con IMC normal, se encontró que las mujeres alcanzarían el percentil 75 (P_75_) a partir de los 49 años y, los hombres, el percentil 90 (P_90_) a partir de los 58 años. Este hallazgo refuerza la importancia de contar con curvas propias del índice cintura-talla, elaboradas con datos de población colombiana, diferenciadas por edad, sexo y etnia, y con puntos de corte originados en la misma población.

En el segundo estudio, realizado por Correa *et al.,* se evaluó población adulta brasilera ^53^ y se encontró que el índice cintura-talla identificaba más individuos con riesgo precoz para la salud que la matriz de combinación entre el IMC y la circunferencia de la cintura. Asimismo, en relación con la prevalencia de hipertensión arterial sistémica ^53^, el índice cintura-talla presentó una capacidad comparable para identificar este riesgo para la salud como puntos de corte 0,52 en hombres y 0,54 en mujeres. En la población colombiana estudiada, estos puntos de corte se ubican en el mismo percentil 75 (P_75_) para ambos sexos, pero para las mujeres a partir de los 56 años y para los hombres a partir de los 49 años. En ambos casos, la detección con estos puntos de corte se daría a una edad tardía para la población colombiana.

En la población adulta colombiana, aún es prematuro y carece de sustento científico plantear que para el índice cintura-talla se pueda utilizar un único punto de corte para ambos sexos. Esto facilitaría la promoción del mensaje de salud pública de “mantenga la circunferencia de su cintura a menos de la mitad de su talla”, tal y como lo han propuesto algunos autores ^48^. Ashwell *et al.* sugirieron que el índice cintura-talla es una herramienta de tamizaje sencilla y rápida, que podría ayudar a superar los debates sobre el uso de diferentes puntos de corte del IMC para evaluar los riesgos de la salud en diferentes poblaciones, por ejemplo, asiáticas y caucásicas ^54^^-^^56^.

Los autores se fundamentan en seis razones:

1. El índice cintura-talla es más sensible que el IMC como alerta precoz de riesgos para la salud.
2. El índice cintura-talla es más barato y fácil de medir y calcular que el IMC, porque no requiere de una báscula.
3. El índice cintura-talla es más fácil de medir y calcular que el IMC si se cuenta con personal entrenado en la técnica de medición.
4. Un valor límite de índice cintura-talla igual a 0,5 indica un mayor riesgo para personas de diferentes grupos étnicos.
5. Los valores límite del índice cintura-talla pueden convertirse en un gráfico fácil de utilizar ^48^.
6. Comunicar mensajes sobre el riesgo para la salud podría ser mucho más sencillo si se utilizaran el mismo índice antropométrico y el mismo mensaje de salud pública durante todo el curso de vida y en todo el planeta ^48^.

Las fortalezas de este estudio son varias:

1. Se trabajó con una muestra representativa del país, con diversidad regional, étnica y socioeconómica.
2. Se delimitaron los análisis a adultos con IMC adecuados, lo cual permitió eliminar comportamientos muy heterogéneos en la cintura por categorías del IMC debido al aumento del promedio del índice cintura-talla, lo que dificultaba diferenciar el indicador entre las diversas categorías de estado nutricional, solapándose entre sí.
3. Las mediciones antropométricas fueron confiables, dado que tanto el uso de métodos como el personal se estandarizaron.

Se generaron curvas de percentiles para representar los datos del índice cintura-talla que servirán para el control del riesgo cardiovascular en población adulta con IMC normal.

Las limitaciones de este estudio están relacionadas con la falta de información sobre antecedentes de enfermedades cardiovasculares o marcadores clínicos, lo cual impidió evaluar la utilidad del punto de corte internacional del índice cintura-talla (> 0,50) como predictor de riesgo de enfermedades no transmisibles en la población estudiada. Asimismo, la base original de la ENSIN 2015 ^2^, no incluyó datos dietarios ni niveles de actividad física para estudiar la composición corporal.

Este estudio presenta curvas de percentiles para el índice de cintura-talla de personas adultas colombianas -con IMC dentro de los parámetros estándar- que muestran algunas diferencias por sexo. Estas curvas pueden ser una herramienta importante para estudios de investigación relacionados con obesidad o riesgo cardiovascular en población adulta.

Por otro lado, los valores obtenidos y las curvas generadas deben ser validados en otros estudios -por ejemplo, de tipo longitudinal- para evaluar su validez externa y utilidad en la práctica clínica, relacionadas con la definición de un punto de corte del índice cintura-talla que ayude a calcular el riesgo de enfermedad no transmisible en el adulto colombiano.
